# Discounting Health Grades for Disparity: The 2021 Wisconsin Population Health and Equity Report Card

**DOI:** 10.5888/pcd20.220301

**Published:** 2023-04-06

**Authors:** Hannah Olson-Williams, Keith P. Gennuso, Marjory L. Givens, David A. Kindig

**Affiliations:** 1 Department of Population Health Sciences, University of Wisconsin, Madison; 2 University of Wisconsin Population Health Institute, University of Wisconsin, Madison

## Abstract

We describe updates to the University of Wisconsin Population Health Institute’s methodology for a state health report card, first described in *Preventing Chronic Disease* in 2010, and the considerations that were weighed in making those updates. These methods have been used since 2006 to issue a periodic report entitled Health of Wisconsin Report Card. The report highlights Wisconsin’s standing among other states and serves as an example for others seeking to measure and improve their population’s health. For 2021, we revisited our approach with an increased emphasis on disparities and health equity, which required many choices about data, analysis, and reporting methods. In this article, we outline the decisions, rationale, and implications of several choices we made in assessing Wisconsin’s health by answering several questions, among them: Who is the intended audience and which measures of length (eg, mortality rate, years of potential life lost) and quality of life (eg, self-reported health, quality-adjusted life years) are most relevant to them? Which subgroups should we report disparities about, and which metric is most easily understood? Should disparities be summarized with overall health or reported separately? Although these decisions are applicable to 1 state, the rationale for our choices could be applied to other states, communities, and nations. Consideration of the purpose, audience, and context for health and equity policy making is important in developing report cards and other tools that can improve the health of all people and places.

**SUMMARY**

**What is already known on this topic?**

The health of US states has been reported in numerous ways. Decisions, many of which are value judgments, are required in communicating and disseminating public health messaging to state-level decision makers.

**What is added by this report?**

We outline the choices made in updating a state health report card evaluating overall health and health disparities in length and quality of life. Although we applied these choices to 1 state, the same rationale could be applied to other states, communities, and nations.

**What are the implications for public health practice?**

Understanding, quantifying, and communicating overall health and health disparities are key to motivating policy and systems change toward improving the health of all people and places.

**MEDSCAPE CME**

In support of improving patient care, this activity has been planned and implemented by Medscape, LLC and *Preventing Chronic Disease*. Medscape, LLC is jointly accredited with commendation by the Accreditation Council for Continuing Medical Education (ACCME), the Accreditation Council for Pharmacy Education (ACPE), and the American Nurses Credentialing Center (ANCC), to provide continuing education for the healthcare team.

Medscape, LLC designates this Journal-based CME activity for a maximum of 1.0 *AMA PRA Category 1 Credit(s)*™. Physicians should claim only the credit commensurate with the extent of their participation in the activity.

Successful completion of this CME activity, which includes participation in the evaluation component, enables the participant to earn up to 1.0 MOC points in the American Board of Internal Medicine's (ABIM) Maintenance of Certification (MOC) program. Participants will earn MOC points equivalent to the amount of CME credits claimed for the activity. It is the CME activity provider's responsibility to submit participant completion information to ACCME for the purpose of granting ABIM MOC credit.

Release date: April 6, 2023; Expiration date: April 6, 2024

**Learning Objectives**

Upon completion of this activity, participants will:
  1. Define health inequity
  2. Assess major changes made to the state health report card described in the current article
  3. Distinguish changes to the health grade methodology employed in the update to the state health report
  4. Identify domains for health disparities in the update to the state health report

Credit Hours – 1.0

EDITOR

Rosemarie Perrin

Editor *Preventing Chronic Disease*

Atlanta, Georgia

AUTHORS

Hannah Olson-Williams, BS

Department of Population Health Sciences

University of Wisconsin

Madison, Wisconsin, USA

Keith P. Gennuso, MS, PhD

University of Wisconsin Population Health Institute

University of Wisconsin

Madison, Wisconsin, USA

Marjory L. Givens, MSPH, PhD

Department of Population Health Sciences

University of Wisconsin Population Health Institute

University of Wisconsin

Madison, Wisconsin, USA

David A. Kindig, MD, PhD

Department of Population Health Sciences

University of Wisconsin Population Health Institute

University of Wisconsin

Madison, Wisconsin, USA

CME AUTHOR

Charles P. Vega, MD

Health Sciences Clinical Professor of Family Medicine

University of California, Irvine School of Medicine

Charles P. Vega, MD, has the following relevant financial relationships:

Consultant or advisor for: GlaxoSmithKline; Johnson & Johnson Pharmaceutical Research & Development, L.L.C.

## Background

In 2006, the University of Wisconsin Population Health Institute developed the methodology for a state health report card that used data for all 50 states to establish grading curves for 2 health outcomes, length of life and quality of life. These outcomes were divided into 4 life stage groups: infants and children, young adults, working-age adults, and older adults. We assigned grades A through F for overall health and health disparities within each life stage by sex, race or ethnicity, socioeconomic status, and community type (1). These methods were used to issue a periodic report to highlight Wisconsin’s standings among other US states. For example, in the 2016 Report Card (https://go.wisc.edu/8ew61p), Wisconsin received a grade of B-minus in overall health and a D for health disparities. For the most recent edition (https://go.wisc.edu/z96or8), “2021 Wisconsin Population Health and Equity Report Card,” motivated by research advances and demand among practitioners for health equity tools in an era of socioeconomic uncertainty (2,3), we recognized the need for better measures of health disparities.

A health disparity is any quantifiable difference in a measurement of health across population subgroups, and a health inequity is a disparity that is systematic, avoidable, unnecessary, unfair, or unjust (4). By recognizing and addressing the causes of disparities that affect groups that are excluded or marginalized in terms of health care access, valuing all individuals and populations equally, recognizing and rectifying historical injustice, and providing resources according to need (5) we can improve population health in an equitable manner and achieve the twin goals of population health: improving the health of the community as a whole and reducing gaps between groups. In this article, we outline the analytic decisions we made in measuring health disparities to help guide the thinking of those considering similar reporting and accountability efforts. These data and analytic decisions could be applied to any state, community, or nation to facilitate concise and easily interpretable public health messaging.

## Considerations for Methods Updates

We encountered several key choice points in updating our state health report card methods. Some of these choices were easy. For example, constraints on available data for secondary analysis meant that we had few choices, and some choices required more nuance. These choices can be summarized into 3 categories: intended purpose and audience, updates to overall health and health disparity grades, and combining overall health and health disparities. We discuss our rationale and the implications of a selection of choices. All choices were made to elevate the issue of health inequities and inform policy and system changes that improve the health of the population overall and decrease disparities in health among population subgroups.

### Intended purpose and audience

The University of Wisconsin Population Health Institute aims to translate research and data for policy and practice. The state health report card was developed as a tool to summarize actionable data and communicate key messages to policy makers and practitioners. Many health-related policies are determined by state-level actors and implemented by local community leaders. As seen during the COVID-19 pandemic, local public health departments, school boards, and city councils make decisions that influence health factors and outcomes. However, these decisions may not affect everyone equally. When available, we chose to disaggregate measures of health by community and social groups in Wisconsin to make our findings as locally applicable as possible.

### Revisiting the overall health grade methodology

In the 2021 update to the state health report card, we simplified methods for grades of overall health to improve interpretability and for easier integration with methods used to capture health disparities. Previous report cards assigned grades by life stage for mortality in the 2 younger life stage groups (infants and children, young adults), and for length of life and quality of life, combined, in the 2 older life stage groups (working-age adults, older adults). These report cards delivered an overall health grade that was a combination of grades across the life stages. To simplify, we removed life stages from the approach and calculated a separate grade for length of life and quality of life. Because of its breadth and simplicity, we chose all-cause, age-adjusted mortality (deaths per 100,000 population, 2015–2019) from the National Center for Health Statistics and CDC WONDER (6) for our measure of length of life. We chose self-reported health status (the percentage of the population reporting fair or poor health in 2020) from the Behavioral Risk Factor Surveillance System (7) as our quality-of-life measure because of its interpretability and legacy as a commonly reported and measured metric of population health (8,9).

Though state grades for length of life and quality of life are the focus of this article, the Wisconsin report card also includes descriptive statistics for several policy action areas that were selected by the Community Resilience and Response Task Force, a partnership among several Wisconsin public health and political agencies. Based on broad cross-sector engagement in Wisconsin, the task force identified the following policy priority areas: quality health care, safe and affordable housing, economic resources for children and families, broadband infrastructure, and civic engagement. Data for these policy areas are not necessarily comparable or relevant across states, and subgroup data can be differentially scarce. Therefore, we chose to use mortality and self-reported health to make the across-state and across-subgroup comparisons necessary for our report card grades. Researchers creating similar reports are encouraged to include data for policy areas with local relevance and availability.

**Figure 1:**
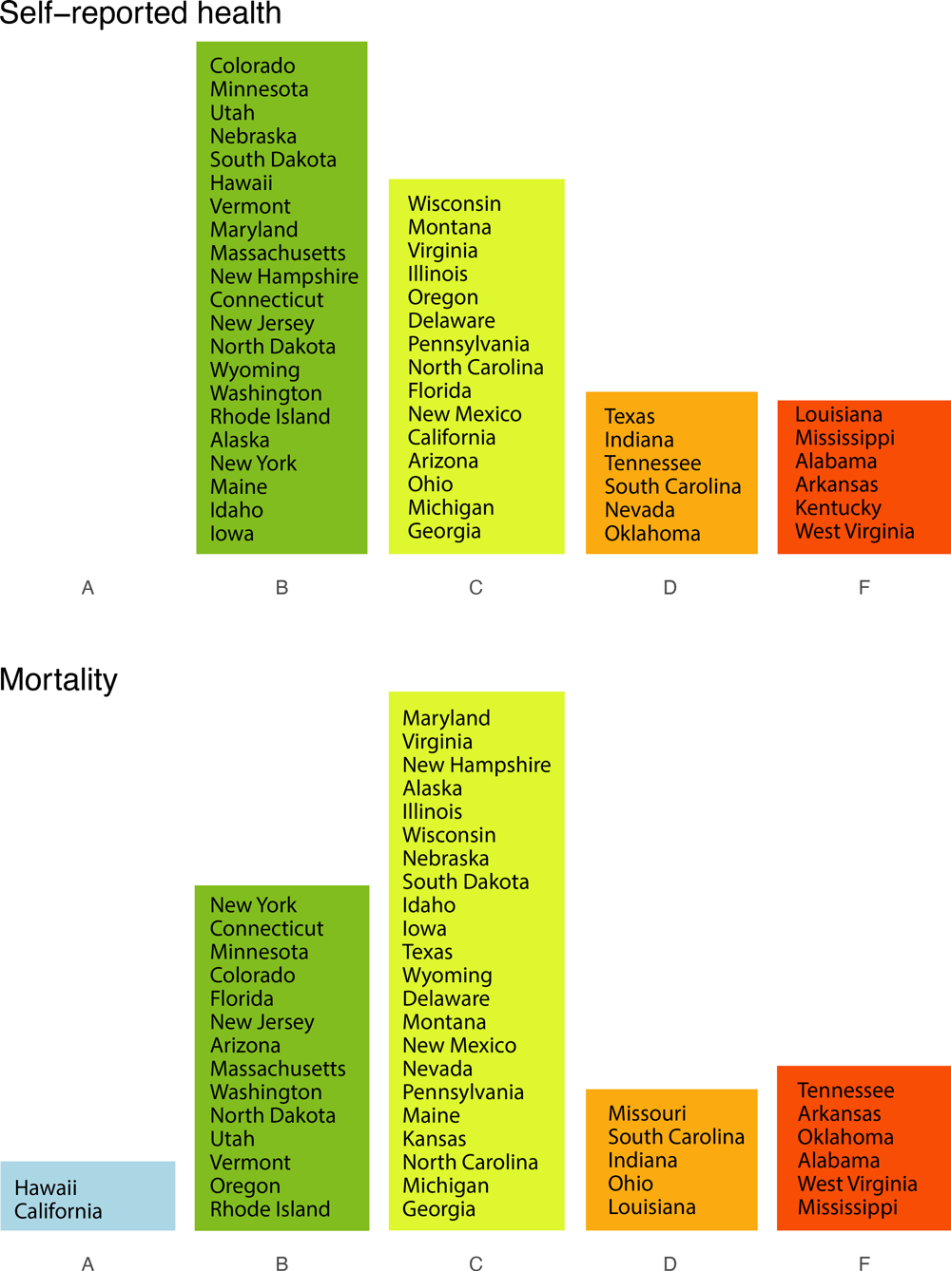
Two geographic grade distributions, self-reported health and mortality, assessed in state health report cards (tools that summarize actionable data and communicate key messages to policy makers and practitioners). Grades are A, B, C, D, and F. Grade cut-offs were assigned by using the national mean and SD of rates of self-reported health (percentage of the population reporting fair or poor health) and mortality (deaths per 100,000 population). States at the top of each column have higher grades and lower rates of mortality or fair/poor health than the states below them. The same cut-offs for SD relative to the national mean were used to assign grades for both distributions: A, >1.5 SD better; B, >0.5 SD to ≤1.5 SD better; C, ≤0.5 SD better and <0.5 SD worse; D, ≥0.5 SD to <1.5 SD worse; and F, ≥1.5 SD worse than the national mean.

**Figure 2:**
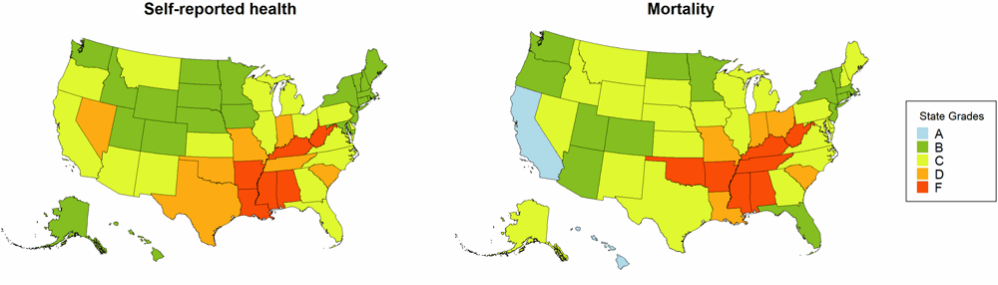
Two choropleth maps of the US showing each state’s letter grade for 2 distributions, self-reported health and mortality, assessed in state health report cards (tools that summarize actionable data and communicate key messages to policy makers and practitioners). Grades are A, B, C, D, and F. Grade cut-offs were assigned by using the national mean and SD of rates of self-reported health (percentage of the population reporting fair or poor health) and mortality (deaths per 100,000 population). The same cut-offs for SD relative to the national mean were used to assign grades for both distributions: A, >1.5 SD better; B, >0.5 to ≤1.5 SD better; C, ≤0.5 SD better and <0.5 SD worse; D, ≥0.5 to <1.5 SD worse; and F, ≥1.5 SD worse than the national mean.

As with previous report cards, grades for length of life and quality of life were assigned according to distribution of *z* scores calculated from the state values for mortality and self-reported health status. We chose to continue using letter grades because they concisely convey complex health data in an interpretable and attention-grabbing manner. Though letter grades result in some information loss, they allow us to convey differences in a simple format familiar to our audience (ie, F is substantially worse than D) without overloading our audience with the complexities of methodology. We described the distributions of state grades by measure (Figure 1). States with values at or above the 93rd percentile (or 1.5 SDs larger than the national mean) received an F grade. Those between the 69th and 93rd percentile received a D; between the 31st and 69th percentile, a C; between the 7th and 31st percentiles, a B; and below the 6th percentile, an A. We also calculated the geographic distributions of state grades across the US (Figure 2). 

### Revisiting the health disparity grade methodology

We revisited methods to determine state-level health disparity grades to incorporate advancements in research related to conceptualizing, calculating, and communicating health disparities (10,11). Previous report cards assigned grades to individual population subgroups by using the same underlying distribution used to calculate overall health grades. For example, in the 2007 State of Wisconsin Report Card, Wisconsin received a grade of F for infant mortality among the African American/Black population subgroup because the rate of 17.6 deaths per 1,000 births was well above the national mean (12). This method has the advantage of providing specific quantification of a health disparity for a population subgroup, but also has significant drawbacks. For example, communicating health disparities by assigning grades to population subgroups could be misinterpreted as a reflection of individual characteristics of each subgroup rather than resulting from the inequitable social and economic conditions that influence health potential. Therefore, we used a health disparity metric that captured the size of the gap between population subgroups within the domains of race and ethnicity, educational attainment, and community type, and grades were assigned to states on the basis of the distribution of the size of those gaps.

### Disparities between whom?

We first had to identify domains for measuring disparities. This choice is, of course, value laden but was also partially limited by data availability. We chose to measure disparities across racial and ethnic identity, educational attainment, and community type because members of these groups are people who may have experienced “othering” (the perception or treatment of individuals or groups as inherently different) (13) that has been harmful to health in both the past and present. We recognize that these are not the only domains in which disparities occur. Unfortunately, finer-grain data to describe the multiple characteristics of identities within population groups are not consistently available across data sources; for example, county-level mortality disaggregated by disability status, sexual orientation, and other intersectional identities (meaning how one can identify as being part of more than 1 marginalized group) (14). For our report card, we sought a matching data set for each domain across both measures of mortality and self-reported health for the population subgroups in the 3 domains for which data were available for the 2 measures of health: self-reported health and mortality (Table 1). 

**Table 1:** Domains, Measures, and Data Sources for Measuring Health Disparities, Grading and Reporting Health and Health Disparities, 2021 Wisconsin State Health Report Card

Characteristic	Self-reported health^a^	Mortality^b^
**Race or ethnicity^c^**	• Non-Hispanic Black • Non-Hispanic White • Non-Hispanic American Indian or Alaska Native • Non-Hispanic Asian • Non-Hispanic Native Hawaiian and Other Pacific Islanders • Non-Hispanic Multiracial • Hispanic	• Non-Hispanic Black • Non-Hispanic White • Non-Hispanic American Indian or Alaska Native • Non-Hispanic Asian • Hispanic
**Educational attainment^d^**	• Less than high school education • High school diploma or equivalent • Some post high school education • Four-year college degree or higher	• Less than high school education • High school diploma or equivalent • Some post high school education • Four-year college degree or higher (3)
**Community type^e^**	• Large urban metro • Large suburban metro • Smaller metro • Rural	• Large urban metro • Large suburban metro • Smaller metro • Rural

a Behavioral Risk Factor Surveillance System, 2020 (7).

b National Center for Health Statistics and CDC WONDER (2015–2019) (6).

c We are bound by data collection and categorization of race and ethnicity according to the US Census Bureau definitions, in adherence with 1997 Office of Management and Budget standards (15). This categorization masks variation within racial and ethnic groups and can hide the historic context that underlies health differences.

d Educational attainment subgroup data were obtained from the National Center for Health Statistics as compiled from data provided by the 57 vital statistics jurisdictions through the Vital Statistics Cooperative Program. The University of Wisconsin Population Health Institute has a data-use agreement in place to use NCHS vital statistics. It should be noted that NCHS vital statistics contain underlying data for the publicly available CDC WONDER mortality estimates, which we used for race and ethnicity and community type.

e Community type is an adaptation of the 2013 National Center for Health Statistics Urban–Rural Classification Scheme for Counties (16). Large urban metro and large suburban metro are central and fringe counties from metropolitan statistical areas (MSAs) of 1 million or more population, respectively. Smaller metro are counties from MSAs of 50,000 to 1 million population and Rural are nonmetropolitan counties.

### Choice of disparity metric

Health disparities can be measured in many different ways (17), each with conceptual and methodologic characteristics that reflect a set of norms and values (18). Measures can differ, for example, regarding the reference value (ie, the value against which subgroups are compared), whether the disparities are weighted by the population size of the subgroups, and whether the disparity is expressed relative to the overall mean (ie, relative vs absolute differences). Of the numerous possibilities for measures, we refined our scope on the basis of intended audience, purpose, and data and analytic capabilities. We then considered the potential implications for action of each option. We settled on between-group variance (BGV) (19) to measure the size of the disparities between subgroups in the 3 domains. BGV fitted our intended purposes because it is a population-weighted measure and reflects absolute disparities from the average health status of the population. BGV is a useful measure of spread when comparing nominal groups, such as racial or ethnic subgroups and community types (17). It was calculated according to the following equation:

*BGV* = ∑*p_j_ (y_j_ - µ)^2^*

where *p_j_*_is_ group *j*’s population size, *y_j_* is group *j*’s average health status, and µ is the average health status of the population.

**Reference value.** We could have applied the BGV method to the data several different ways, depending on the nature of the intended comparison. By comparing subgroups state by state within domains and using the state mean for µ, we were able to ask, for example, “How healthy are rural counties in Wisconsin relative to the average Wisconsin county?” We found alternatives, such as using the national mean for µ, which would ask, “How healthy are rural counties in Wisconsin relative to the average US county?” or a national subgroup mean for µ, which would ask, “How healthy are rural counties in Wisconsin relative to the average rural county in the US?” These alternatives have merit but were less relevant for state and local policy makers to act on. For example, using the national mean for µ would compare the health of subgroups in Wisconsin to a national average that includes states with no indoor smoking bans, where abortion is more accessible (20), and that encompass high-density metropolitan areas. When national-level comparisons are made, important state-level policy and contextual factors cannot be accounted for.

**Population-weighting.** BGV weights the size of the difference between each subgroup and the reference value by the population proportion of the subgroup. Without added population weights, differences in health across all subgroups would be considered equally, regardless of population size. A state with a very small subgroup that experiences disparate health would be given the same disparity rating as a state with a very large subgroup that experiences the same level of disparate health. Since our intended audience is policy makers, we considered population weighting to be a desirable characteristic because it is conducive to understanding the types of per capita investments that would be necessary to reduce or eliminate the disparities. This choice does, however, place emphasis on the health status of the dominant population group for the state, for example, the White population in Wisconsin potentially outweighing large disparities in small population subgroups, such as American Indian or Alaska Native populations.

**Absolute versus relative disparities.** Absolute disparity is the simple difference between a group rate and the reference point and is reported in the units of the underlying data. Relative disparity is the same difference but expressed as a percentage of the reference point and is unitless (21). BGV is considered a measure of absolute disparity because the sum of the squared differences is not made relative to the reference value (ie, the state mean). Although absolute and relative measures convey different but important information about differences in health in a community, population health interventions often rely on an understanding of the absolute burden of disease (22). Thus, when faced with choosing a single measure of disparity, we prioritized absolute disparity. In addition, our ultimate objective of adjusting each state’s overall grades for mortality and self-reported health based on the size of health disparities also favored the use of an absolute disparity measure. Using a relative measure of disparity would have taken the state mean value into consideration twice, once during the calculation of the relative disparity measure and again during the adjustment.

### Combining overall health and health disparities

Because we wanted to present a simple and easy-to-interpret measure of health disparity in the context of our overall health outcome grades, we calculated mortality and self-reported health, adjusted for subgroup disparities as measured by BGV. Our method of disparity-adjusted grades allowed us to maintain a familiar format for our audience with overall state grades while presenting a new grading scheme under which no states could have received improved grades — that is, all grades worsened because of disparities and disparities are problematic in all states.

We calculated a total of 6 disparities grades for each state, 1 for each of the 3 domains (educational attainment, race or ethnicity, and community type) for both mortality and self-reported health. After calculating the BGV for each domain and health outcome, we then rescaled BGV to be between 0 and 1, so that states with high levels of disparity had values close to 1 and states with low levels of disparity had values near 0. For each state, we multiplied the state mean mortality rate and the state mean prevalence of fair or poor health by 1+rescaled BGV. We then used these “adjusted” mortality and self-reported health rates to recalculate state *z* scores by using the mean and standard deviation of all 50 states. For each domain, we then standardized and assigned grades by using the same *z* score cut-offs used in calculating overall grades.

By calculating a separate disparity grade for each domain, we aimed to make our grades more interpretable to a general audience and more actionable for a policy maker audience. For instance, the state of Wisconsin received a C grade for self-reported health. After adjustment for disparities by community type, the grade remained a C. However, after adjustment for disparities by race and ethnicity and educational attainment, Wisconsin received grades of D and F, respectively, for self-reported health. This indicates that Wisconsin should focus not only on improving its population’s overall self-reported health but also on reductions in disparities between racial and ethnic groups and groups with different levels of educational attainment (Table 2).

**Table 2:** The 2021 Wisconsin State Health Report Card Overall and Disparity-Adjusted Health Grades

Category	Health grade^a^	
	Mortality	Self-reported health
**Wisconsin’s health grades**	C	C
Adjusted for community type disparities	D	C
Adjusted for racial or ethnic disparities	D	D
Adjusted for educational disparities	F	F

a Letter grades are intended to be interpreted like traditional school report card grades where a C indicates fair or average performance, a D indicates poor performance, and an F indicates failure.

### Commentary

To assess the twin goals of population health — improving the health of the population overall and reducing disparities between subgroups — many value-laden choices are required. Establishing an intended audience and purpose, deciding which subgroups to center measurements on, and determining how best to display and communicate findings are just a few. These choices must be considered carefully when reporting the health of people and places. Each of these choice points has meaningful tradeoffs with implications for the actions they could inspire. The methods outlined here are restricted by available data for secondary analysis. We are bound by data collection and categorization of race and ethnicity according to the US Census Bureau definitions, in adherence to the 1997 Office of Management and Budget standards (15). Additionally, many identities, circumstances, and structures could or should be the focus of policy makers. However, because national data sets do not capture increasingly diverse population subgroups and phenomena, report cards like ours cannot measure their relative health and use data-to-action tools to advocate for them. Of particular importance, and missing from the analyses we outline in this article, are intersectional identities, that is, people identifying with more than 1 group. We chose to measure disparities across racial and ethnic identities, educational attainment, and community type because these groups represent people who may have experienced othering that has been harmful to health in both the past and present. However, additional context is needed to precisely name variation in health and opportunity within subgroups and how these group-level differences may affect individuals. Current lack of granular data on groups that have experienced othering is an inequity in and of itself. Without data, we cannot understand or advocate for the health of all individuals. This is an avoidable structural problem.

When formulating our approach, we relied heavily on the precedent set by previous report cards. We believed it important to create a data-to-action tool that felt familiar to previous report cards while adding novel disparity metrics. However, because the methodology used to create estimates for self-reported health have changed in recent years, comparisons over time should be made with caution. Therefore, we chose to present data from a single point in time. The underlying data and grading methods for the 2021 report card are not comparable to previous versions of the report card and not useful for tracking progress or effects of policy changes.

Our methods were inspired by the Social Determinants of Health framework (23), placing emphasis on how place (or state, in the case of our analyses) may result in differences in length and quality of life across social groups. We also draw from the Fundamental Cause Theory of Health (24), which advocates for the replacement of individual-level interventions with broad, society-level interventions for improving the health of society as a whole and removing social conditions as a fundamental cause of disease. In line with our goals with the report card, these theories emphasize both the urgency and nuance required to work toward the elimination of health disparities.

Although we applied the decisions outlined in this article to 1 state, the rationale for our decisions could be applied to other states, communities, and nations. Understanding, quantifying, and communicating overall health and health disparities is an important step in motivating policy change toward improving the health of all people and places.

## Post-Test Information

To obtain credit, you should first read the journal article. After reading the article, you should be able to answer the following, related, multiple-choice questions. To complete the questions (with a minimum 75% passing score) and earn continuing medical education (CME) credit, please go to http://www.medscape.org/journal/pcd. Credit cannot be obtained for tests completed on paper, although you may use the worksheet below to keep a record of your answers.

You must be a registered user on http://www.medscape.org. If you are not registered on http://www.medscape.org, please click on the “Register” link on the right hand side of the website.

Only one answer is correct for each question. Once you successfully answer all post-test questions, you will be able to view and/or print your certificate. For questions regarding this activity, contact the accredited provider, CME@medscape.net. For technical assistance, contact CME@medscape.net. American Medical Association’s Physician’s Recognition Award (AMA PRA) credits are accepted in the US as evidence of participation in CME activities. For further information on this award, please go to https://www.ama-assn.org. The AMA has determined that physicians not licensed in the US who participate in this CME activity are eligible for AMA PRA Category 1 Credits™. Through agreements that the AMA has made with agencies in some countries, AMA PRA credit may be acceptable as evidence of participation in CME activities. If you are not licensed in the US, please complete the questions online, print the AMA PRA CME credit certificate, and present it to your national medical association for review.

## Post-Test Questions

### Study Title: Discounting Health Grades for Disparity: the 2021 Wisconsin Population Health and Equity Report Card

#### CME Questions

1. Which of the following pairs of characteristics best helps define a health inequity?
  - It occurs at random and is unfair or unjust
  - It is avoidable and systematic
  - Is based on race/ethnicity and affects minority communities
  - It is inevitable and a product of systemic bias
2. Which of the following was a major update to the authors’ previous state health reports described in the current paper?
  - Combining overall health and health disparities
  - Specific focus on social determinants of health
  - Benchmarking against the previous 5 years of state data
  - Analysis of impact of chronic disease on disability rates
3. What was the main change to the health grade methodology in the current study?
  - Stratification by life stages
  - Combining length-of-life and quality-of-life data
  - Inclusion of disability data
  - Removing life stages but separating length-of-life and quality-of-life data
4. Health disparities were measured across which variables in the revision to the Health of Wisconsin Report Card?
  - Sexual orientation, veteran status, place of residence
  - Socioeconomic status, relationship status, aboriginal status
  - Racial/ethnic identity, educational attainment, community type
  - Disability, gender, age

## References

[1] Booske BC , Rohan AM , Kindig DA , Remington PL . Grading and reporting health and health disparities. Prev Chronic Dis 2010;7(1):A16. 20040231 PMC2811511

[2] Gupta AH . Keys to an equitable recovery: better data and ‘trusted messengers’. The New York Times. Published January 15, 2021. Accessed August 14, 2022. https://www.nytimes.com/2021/01/14/us/covid-biden-race-gender-healthcare.html

[3] Raymond N , Chambers A , Polatty D , Khoshnood K , Xu R . Operationalizing equity during local pandemic response (submission eclinm-d-21-00233R1). EClinicalMedicine 2021;36:100937. 10.1016/j.eclinm.2021.100937 34142072 PMC8187830

[4] Whitehead M . The concepts and principles of equity and health. Int J Health Serv 1992;22(3):429–45. 10.2190/986L-LHQ6-2VTE-YRRN 1644507

[5] Braveman P . What are health disparities and health equity? We need to be clear *.* Public Health Rep 2014;129(suppl 2):5–8. 10.1177/00333549141291S203 24385658 PMC3863701

[6] Centers for Disease Control and Prevention. CDC WONDER. Accessed August 14, 2022. https://wonder.cdc.gov/

[7] Centers for Disease Control and Prevention. PLACES: Local data for better health, Place Data 2020 release. Accessed August 14, 2022. https://chronicdata.cdc.gov/500-Cities-Places/PLACES-Local-Data-for-Better-Health-Place-Data-202/q8xq-ygsk

[8] Lopez AD . Summary measures of population health: concepts, ethics, measurement and applications. World Health Organization; 2002.

[9] Bowling A . Measuring Health. McGraw-Hill Education (UK); 2004.

[10] Penman-Aguilar A , Talih M , Huang D , Moonesinghe R , Bouye K , Beckles G . Measurement of health disparities, health inequities, and social determinants of health to support the advancement of health equity. J Public Health Manag Pract 2016;22 (suppl 1):S33–S42. 10.1097/PHH.0000000000000373 26599027 PMC5845853

[11] United Nations Development Program, Human Development Reports. Inequality-adjusted human development index (IHDI). Accessed May 17, 2022. https://hdr.undp.org/en/content/inequality-adjusted-human-development-index-ihdi

[12] Hatchell K , Handrick L , Pollock E , Timberlake K . Health of Wisconsin. Summary grades. Report card 2016. Accessed August 14, 2022. https://uwphi.pophealth.wisc.edu/wp-content/uploads/sites/316/2018/01/Report-Card-2016.pdf

[13] Canales MK . Othering: Toward an understanding of difference. ANS Adv Nurs Sci 2000;22(4):16–31. 10.1097/00012272-200006000-00003 10852666

[14] Crenshaw K . Demarginalizing the intersection of race and sex: a Black feminist critique of antidiscrimination doctrine, feminist theory and antiracist politics. Routledge; 1991.

[15] Office of Management and Budget (OMB) Revisions to the Standards for the Classification of Federal Data on Race and Ethnicity. Federal Register 1997;62(210):58782. Accessed September 15, 2022. https://www.govinfo.gov/content/pkg/FR-1997-10-30/pdf/97-28653.pdf

[16] Ingram DD , Franco SJ . 2013 NCHS urban–rural classification scheme for counties. Vital Health Stat 2 2014;(166):1–73. 24776070

[17] Harper S , Lynch J . Methods for measuring cancer disparities: using data relevant to Healthy People 2010 Cancer-Related Objectives. APA PsycNet. 2010. Accessed August 14, 2022. 10.1037/e606392012-001

[18] Givens ML , Gennuso KP , Pollock EA , Johnson SL . Deconstructing inequities – transparent values in measurement and analytic choices. N Engl J Med 2021;384(19):1861–65. 10.1056/NEJMms2035717 33979494

[19] National Cancer Institute. Between-Group Variance (BGV). Accessed August 14, 2022. https://seer.cancer.gov/help/hdcalc/inference-methods/individual-level-survey-sample-1/measures-of-absolute-disparity/between-group-variance-bgv

[20] Addante AN , Eisenberg DL , Valentine MC , Leonard J , Maddox KEJ , Hoofnagle MH . The association between state-level abortion restrictions and maternal mortality in the United States, 1995–2017. Contraception 2021;104(5):496–501. 10.1016/j.contraception.2021.03.018 33781761

[21] King NB , Harper S , Young ME . Use of relative and absolute effect measures in reporting health inequalities: structured review. BMJ 2012;345:e5774. 10.1136/bmj.e5774 22945952 PMC3432634

[22] Mabry PL , Olster DH , Morgan GD , Abrams DB . Interdisciplinarity and systems science to improve population health: a view from the NIH Office of Behavioral and Social Sciences Research. Am J Prev Med 2008;35(2 suppl):S211–24. 10.1016/j.amepre.2008.05.018 18619402 PMC2587290

[23] World Health Organization. A Conceptual Framework for Action on the Social Determinants of Health. Discussion paper for the commission on social determinants of health; 2010. Accessed August 14, 2022. https://www.who.int/publications/i/item/9789241500852

[24] Link BG , Phelan J . Social conditions as fundamental causes of disease. J Health Soc Behav 1995;35(Spec No):80–94. 10.2307/2626958 7560851

